# Supplementary material for: Factors associated with female infertility in Ethiopia: A systematic review and meta-analysis
Source: PLoS One. 2025 May 16;20(5):e0323181. doi: 10.1371/journal.pone.0323181 (PMC12083798; doi:10.1371/journal.pone.0323181)
Supplement: S2 Table — (DOCX) [file pone.0323181.s002.docx]

**S2 Table:** Searching strategies for some databases and search engine to assess the factors associated with female infertility in Ethiopia

| **Databases and** **search engine** | **Searching terms** | **Number of studies** | **Searching period** |
| --- | --- | --- | --- |
| PubMed | (("Infertility, Female"[MeSH] OR "female infertility"[All Fields] OR "infertility in women"[All Fields] OR "women's infertility"[All Fields]) AND ("Risk Factors"[MeSH] OR "determinants"[All Fields] OR "causes"[All Fields] OR "factors"[All Fields] OR "risk factors"[All Fields]) AND ("Ethiopia"[MeSH] OR Ethiopia[All Fields])) | 10 | January 1, 2000, to March 20, 2024 |
| google scholar (Advanced search) | allintitle: determinants and " infertility " and Ethiopia | 4 |  |
| African Journals Online (AJOL) | ("female infertility" OR "infertility in women" ) AND ("determinants" OR "risk factors") AND ("Ethiopia" ) | 37 |  |
| DOAJ | determinants and female infertility and Ethiopia | 1 |  |
| EBSCO | ("female infertility" OR "women's infertility") AND Ethiopia | 5 |  |
| Gray literature (specifically unpublished studies) |  | 2 |  |
| Total searched articles |  | 59 |  |
| Finally, fulfill the eligibility criteria for our review |  | 6 |  |
