# Supplementary material for: Factors associated with female infertility in Ethiopia: A systematic review and meta-analysis
Source: PLoS One. 2025 May 16;20(5):e0323181. doi: 10.1371/journal.pone.0323181 (PMC12083798; doi:10.1371/journal.pone.0323181)
Supplement: S4 Table — (DOCX) [file pone.0323181.s004.docx]

**S4 Table: Shows all studies identified in the literature search, including those that were excluded from the analyses**

| **No** | **Study reference** | **Included (Yes/no)** | **Reason for exclusion** | **Access details for unpublished** |
| --- | --- | --- | --- | --- |
| 1 | Mekdes et al. Magnitude of infertility and associated factors among women attending selected public hospitals in Addis Ababa, Ethiopia: a cross-sectional study. BMC Women's Health. 2022 2022/01/11;22(1):11. | Yes |  |  |
| 2 | Rehima et al. Determinants of Primary Infertility Among Married Women Attending Obstetrics and Gynecology Speciality Centers at Adama Town, Oromia, Ethiopia. American Journal of Life Sciences. 2022;10 (1):10-20. | Yes |  |  |
| 3 | Zerihun et al. Determinants of Infertility Among Married Women Attending Health Facilities in Bahirdar City, North West Ethiopia 2021 | Yes |  | <https://scholar.google.com/scholar?hl=en&as_sdt=0%2C5&q=Determinants+of+Infertility+Among+Married+Women+Attending+Health+Facilities+in+Bahirdar+City%2C+North+West+Ethiopia&btnG>= |
| 4 | Hailegebriel et al. Risk factors of infertility among women attending infertility clinics at st. Paul’s hospital millennium medical college in addis ababa 2022. | Yes |  | <https://scholar.google.com/scholar?hl=en&as_sdt=0%2C5&q=.+Risk+factors+of+infertility+among+women+attending+infertility+clinics+at+st.+Paul%E2%80%99s+hospital+millennium+medical+college+in+addis+ababa+&btnG>= |
| 5 | Desalegn et al. Determinants of Infertility among Married Women Attending Dessie Referral Hospital and Dr. Misganaw Gynecology and Obstetrics Clinic, Dessie, Ethiopia. International Journal of Reproductive Medicine. 2020 2020/03/27;2020: 1540318. | Yes |  |  |
| 6 | Nanati et al. The prevalence of infertility and factors associated with infertility in Ethiopia: Analysis of Ethiopian Demographic and Health Survey (EDHS). PLOS ONE. 2023;18(10):e0291912. | Yes |  |  |
| 7 | Teklemicheal AG, Kassa EM, Weldetensaye EK. Prevalence and correlates of infertility related psychological stress in women with infertility: a cross-sectional hospital based survey. BMC psychology. 2022 Apr 7;10(1):91. | No | Different outcomes of interest |  |
| 8 | HANA G. PREVALENCE OF ABNORMAL SEMEN ANALYSIS AND ASSOCIATED FACTORS AMONG MALE PARTNER OF COUPLES WHO CAME FOR INFERTILITY SCREENING IN SELECTED INFERTILITY CENTER IN ADDIS ABEBA, ETHIOPIA, 2023 (Doctoral dissertation). | No | Different outcomes of interest |  |
| 9 | Ahmadi K, Moosavian M, Mardaneh J, Pouresmaeil O, Afzali M. Prevalence of Chlamydia trachomatis, Ureaplasma parvum and Mycoplasma genitalium in infertile couples and the effect on semen parameters. Ethiopian Journal of Health Sciences. 2023 Jan 1;33(1). | No | Irrelevant or unrelated titles |  |
| 10 | Kitilla T. Infertility investigation: socio-demographic characteristics and dropouts of infertile women at Family Guidance Association of Ethiopia (FGAE). Ethiopian Journal of Health Development. 2000;14(2):127-34. | No | Different outcomes of interest |  |
| 11 | Sedlander E, Yilma H, Emaway D, Rimal RN. If fear of infertility restricts contraception use, what do we know about this fear? An examination in rural Ethiopia. Reproductive health. 2022 Jun 13;19(Suppl 1):57. | No | Irrelevant or unrelated titles |  |
| 12 | Bayouh FA. Socio-cultural perceptions of infertility and their implications: a study of women experiencing childlessness in South Gondar, Ethiopia (Master's thesis, The University of Bergen). | No | Different outcomes of interest |  |
| 13 | Yigeremu M, Kebede T, Getachew A, Biruk M. Sonohysterography Assessment of the Structural Abnormalities of the Uterus in Women with Infertility in Ethiopia. Ethiopian Medical Journal. 2024;62(1):1. | No | Different outcomes of interest |  |
| 14 | Admassie D, Negatuy Y. Evaluation of the fallopian tubes in infertile women by hysterosalpingography in Tikur Anbessa Hospital, Addis Ababa, Ethiopia. Int J Nurs Midwifery. 2011 Nov;11:178-81. | No | Different outcomes of interest |  |
| 15 | Meskelu J, Berhane Y. Experiences of women with infertility and their treatment seeking practices: a qualitative study. Ethiopian Journal of Reproductive Health. 2018 Dec 7;10(4). | No | Different outcomes of interest |  |
| 16 | Tilahun T, Oljira R, Getahun A. Pattern of semen analysis in male partners of infertile couples in Western Ethiopia: Retrospective cross-sectional study. SAGE Open Medicine. 2022 Mar;10:20503121221088100. | No | Different outcomes of interest |  |
| 17 | Gebremedhin S, Betre M. Level and differentials of fertility in Awassa town, Southern Ethiopia. African journal of reproductive health. 2009;13(1). | No | Irrelevant or unrelated titles |  |
| 18 | Alene GD, Worku A. Differentials of fertility in North and South Gondar zones, northwest Ethiopia: A comparative cross-sectional study. BMC Public Health. 2008 Dec;8:1-2. | No | Irrelevant or unrelated titles |  |
| 19 | Egbe TO, Nana-Njamen T, Elong F, Tchounzou R, Simo AG, Nzeuga GP, Njamen Nana C, Manka’a E, Tchente Nguefack C, Halle-Ekane GE. Risk factors of tubal infertility in a tertiary hospital in a low-resource setting: a case-control study. Fertility Research and Practice. 2020 Dec;6:1-9. | No | Different outcomes of interest |  |
| 20 | Deribe K, Anberbir A, Regassa G, Belachew T, Biadgilign S. Infertility: Perceived causes and experiences in rural southwest Ethiopia. Age (N= 223*). 2007 Jul;20(2):0-9. | No | Different outcomes of interest |  |
| 21 | Ombelet W. Reproductive healthcare systems should include accessible infertility diagnosis and treatment: an important challenge for resource-poor countries. International Journal of Gynecology & Obstetrics. 2009 Aug 1;106(2):168-71. | No | Irrelevant or unrelated titles |  |
| 22 | Alemayehu T, Haider J, Habte D. Determinants of adolescent fertility in Ethiopia. Ethiopian Journal of Health Development. 2010;24(1). | No | Irrelevant or unrelated titles |  |
| 23 | Kitilla T. Semen quality of suspected infertile Ethiopians at Family Guidance Association of Ethiopia (FGAE) Central Clinic, Addis Ababa: a retrospective review. Ethiopian Medical Journal. 2002 Oct 1;40(4):325-34. | No | Different outcomes of interest |  |
| 24 | Tadesse E, Teshome M, Amsalu A, Shimelis T. Genital Chlamydia trachomatis infection among women of reproductive age attending the gynecology clinic of Hawassa University Referral Hospital, Southern Ethiopia. PloS one. 2016 Dec 22;11(12):e0168580. | No | Irrelevant or unrelated titles |  |
| 25 | Kitilla T. Tubo-peritoneal infertility: comparision of pre-operative hysterosalpingography and laparotomy findings (Tikur Anbessa Hospital, 1995-2002). Ethiopian Medical Journal. 2006 Apr 1;44(2):167-74. | No | Irrelevant or unrelated titles |  |
| 26 | Roba KT, Hassen TA, Wilfong T, Legese Alemu N, Darsene H, Zewdu G, Negese T, Yifru B, Mohammed E, Raru TB. Association of undernutrition and female infertility in East Africa: Finding from multi-country demographic and health surveys. Frontiers in Global Women's Health. 2022 Dec 15;3:1049404. | No | Irrelevant or unrelated titles |  |
| 27 | Araoye MO. Epidemiology of infertility: social problems of the infertile couples. West Afr J Med. 2003 Jun;22(2):190-6. doi: 10.4314/wajm.v22i2.27946. PMID: 14529236. Development Research. 2017;39(1):35-62. | No | Irrelevant or unrelated titles |  |
| 28 | Gure T. Infertility caused by an unnoticed intrauterine contraception device: Case series. SAGE Open Med Case Rep. 2023 May 23;11:2050313X231174100. doi: 10.1177/2050313X231174100. PMID: 37250820; PMCID: PMC10214076. | No | Irrelevant or unrelated titles |  |
| 29 | Tsegazeab H. Prevalence of male factor infertility and associated factors in couples seeking fertility Care at St. Paul’s Hospital Millennium Medical College, Addis Ababa, Ethiopia (Doctoral dissertation). | No | Different outcomes of interest |  |
| 30 | Zurlo MC, Cattaneo Della Volta MF, Vallone F. Infertility-Related Stress and Psychological Health Outcomes in Infertile Couples Undergoing Medical Treatments: Testing a Multi-dimensional Model. J Clin Psychol Med Settings. 2020 Dec;27(4):662-676. doi: 10.1007/s10880-019-09653-z. PMID: 31471847. | No | Irrelevant or unrelated titles |  |
| 31 | Moges N. Follow-Up of Infertility Problems of Indigenous Zebu and Crossbred Dairy Cows in Dairy Farms in Gondar, North West Ethiopia. European Journal of Applied Sciences. 2014;6(4):72-7. | No | Irrelevant or unrelated titles |  |
| 32 | Reda MG, Bune GT, Shaka MF. Research Article Epidemiology of High Fertility Status among Women of Reproductive Age in Wonago District, Gedeo Zone, Southern Ethiopia: A Community-Based Cross-Sectional Study. | No | Irrelevant or unrelated titles |  |
| 33 | Haile F, Gebeyehu S, Abdulkadir H, Gizachew Y, Hailu M. Determinants of infertility among married women who attend gynecologic unit at health facilities of Gamo Zone and South Omo Zone, Southern Ethiopia: A case control study. | No | Irrelevant or unrelated titles |  |
| 34 | Hassa H, Ayranci U, Unluoglu I, Metintas S, Unsal A. Attitudes to and management of fertility among primary health care physicians in Turkey: An epidemiological study. BMC Public Health. 2005 Dec;5:1-8. | No | Irrelevant or unrelated titles |  |
| 35 | Woodall PA, Kramer MR. Schistosomiasis and Infertility in East Africa. Am J Trop Med Hyg. 2018 Apr;98(4):1137-1144. doi: 10.4269/ajtmh.17-0280. Epub 2018 Jan 4. PMID: 29313478; PMCID: PMC5928810. | No | Irrelevant or unrelated titles |  |
| 36 | Roba KT, Hassen TA, Wilfong T, Legese Alemu N, Darsene H, Zewdu G, Negese T, Yifru B, Mohammed E, Raru TB. Association of undernutrition and female infertility in East Africa: Finding from multi-country demographic and health surveys. Front Glob Womens Health. 2022 Dec 15;3:1049404. doi: 10.3389/fgwh.2022.1049404. PMID: 36589148; PMCID: PMC9797807. | No | Irrelevant or unrelated titles |  |
| 37 | Kassa EM, Kebede E. Time-to-Pregnancy and Associated Factors among Couples with Natural Planned Conception in Addis Ababa, Ethiopia. Afr J Reprod Health. 2018 Sep;22(3):33-42. doi: 10.29063/ajrh2018/v22i3.4. PMID: 30381930. | No | Irrelevant or unrelated titles |  |
| 38 | Kitilla T. Hysterosalpingography in the evaluation of infertility: a five years review. (FGAE, 2001 -5). Ethiop Med J. 2010 Oct;48(4):267-75. PMID: 21280428. | No | Irrelevant or unrelated titles |  |
| 39 | Eggert J, Li X, Sundquist K. Country of birth and hospitalization for pelvic inflammatory disease, ectopic pregnancy, endometriosis, and infertility: a nationwide study of 2 million women in Sweden. Fertil Steril. 2008 Oct;90(4):1019-25. doi: 10.1016/j.fertnstert.2007.07.1345. Epub 2007 Sep 19. PMID: 17880949. | No | Irrelevant or unrelated titles |  |
| 40 | Gaym A, Getaneh W, B/Tsion Y. Primary fallopian tube carcinoma (PFTC) comorbidity with infertility and bilateral hydrosalpinx. Ethiop Med J. 2007 Jan;45(1):95-102. PMID: 17642164. | No | Irrelevant or unrelated titles |  |
| 41 | Eyasu Mesfin Kassa and Eskinder Kebede: Time-to-Pregnancy and Associated Factors among Couples with  Natural Planned Conception in Addis Ababa, Ethiopia | No | Irrelevant or unrelated titles |  |
| 42 | Siferih M, Gebre T, Hunduma F, Abebe A, Gebremichael A, Sewunet H, Shibabaw T. Review of Asherman syndrome and its hysteroscopic treatment outcomes: experience in a low-resource setting. BMC Womens Health. 2024 Feb 7;24(1):99. doi: 10.1186/s12905-024-02944-0. PMID: 38326846; PMCID: PMC10848492. | No | Irrelevant or unrelated titles |  |
| 43 | Pinsky AN, Steenbergh K, Boyd HM, Michael B, Bekele D, Kobernik E, Gebremedhin LT, Mmeje O. Healthcare provider attitudes regarding the provision of assisted reproductive services for HIV-affected couples in Addis Ababa, Ethiopia. Int J Gynaecol Obstet. 2018 Apr;141(1):45-51. doi: 10.1002/ijgo.12426. Epub 2018 Jan 28. PMID: 29243253. | No | Irrelevant or unrelated titles |  |
| 44 | Kassaye K, Anberbir A, Regassa G, Belachew T, Biadgilign S. Infertility: perceived causes and experiences in rural southwest Ethiopia. | No | Irrelevant or unrelated titles |  |
| 45 | Mussie R. Gender Differences in Experiences with and Adjustments to Infertility: The Case of Infertility Patients at Family Guidance Association of Ethiopia. Ethiopian Journal of Development Research. 2017;39(1):35-62. | No | Irrelevant or unrelated titles |  |
| 46 | Meskelu J, Berhane Y. Experiences of women with infertility and their treatment seeking practices: a qualitative study. Ethiopian Journal of Reproductive Health. 2018 Dec 7;10(4). | No | Irrelevant or unrelated titles |  |
| 47 | Tilson D, Larsen U. Divorce in Ethiopia: the impact of early marriage and childlessness. J Biosoc Sci. 2000 Jul;32(3):355-72. doi: 10.1017/s0021932000003552. PMID: 10979229.) | No | Irrelevant or unrelated titles |  |
| 48 | Tamrakar SR, Bastakoti R. Determinants of Infertility in Couples. J Nepal Health Res Counc. 2019 Apr 28;17(1):85-89. doi: 10.33314/jnhrc.1827. PMID: 31110383. | No | Irrelevant or unrelated titles |  |
| 49 | Asfaw LS, Alene GD. Marital dissolution and associated factors in Hosanna, Southwest Ethiopia: a community-based cross-sectional study. BMC Psychol. 2023 Jan 25;11(1):20. doi: 10.1186/s40359-023-01051-3. PMID: 36694249; PMCID: PMC9875535. | No | Irrelevant or unrelated titles |  |
| 50 | Ron-El R, Weinraub Z, Langer R, Bukovsky I, Caspi E. The importance of ultrasonography in infertile women with "forgotten" intrauterine contraceptive devices. Am J Obstet Gynecol. 1989 Jul;161(1):211-2. doi: 10.1016/0002-9378(89)90268-8. PMID: 2665497. | No | Irrelevant or unrelated titles |  |
| 51 | Gnoth C, Godehardt E, Frank-Herrmann P, Friol K, Tigges J, Freundl G. Definition and prevalence of subfertility and infertility. Hum Reprod. 2005 May;20(5):1144-7. doi: 10.1093/humrep/deh870. Epub 2005 Mar 31. PMID: 15802321. | No | Irrelevant or unrelated titles |  |
