# Supplementary material for: Factors associated with female infertility in Ethiopia: A systematic review and meta-analysis
Source: PLoS One. 2025 May 16;20(5):e0323181. doi: 10.1371/journal.pone.0323181 (PMC12083798; doi:10.1371/journal.pone.0323181)
Supplement: Attachment — Submitted filename: Response to Reviewers.pdf [file pone.0323181.s006.pdf]

<u>Authors' Point-by-Point Response to the reviewers' and editors' Reports</u>

Title: Determinants of female infertility in Ethiopia: a systematic review and meta-analysis

Journal: PLOS ONE

Article type: Systematic review and Meta-analysis

Submission ID: PONE-D-24-14492

<u>Point by point response to academic editor's comment</u>

Dear Dr. Phakkharawat Sittiprapaporn (Academic Editor),

We are grateful for your consideration of this manuscript, and we also very much appreciate your suggestions, which have been very helpful in improving the quality and impact of the manuscript. We have addressed all the concerns in a point by point manner and have accordingly revised the manuscript. We have highlighted the response in the response letter as well as in the revised manuscript

Comment: Please ensure that your manuscript meets PLOS ONE's style requirements, including those for file naming. The PLOS ONE style templates can be found at https://journals.plos.org/plosone/s/file?id=wjVg/PLOSOne_formatting_sample_main_body.pdf and https://journals.plos.org/plosone/s/file?id=ba62/PLOSOne_formatting_sample_title_authors_affiliations.pdf

Comment: Please include in the Methods section details of when the literature search was conducted for this study (currently this is only mentioned in the S1 Table)

Response: thank you very much for your constructive feedback. We have revised this section as per your suggestion. The search period spanned from January 1, 2000, to March 20, 2024. Detailed information about the literature search strategies is provided in Supplementary Table S2 (S2 Table).

Comment: We note that your Data Availability Statement is currently as follows: "The authors confirm that the data supporting the findings of this study are available within the manuscript and its supplementary materials. Furthermore, the corresponding author will be contacted if someone wants to access the data for this study."

Please confirm at this time whether or not your submission contains all raw data required to replicate the results of your study. Authors must share the "minimal data set" for their submission. PLOS defines the minimal data set to consist of the data required to replicate all study findings reported in the article, as well as related metadata and methods (https://journals.plos.org/plosone/s/data-availability#loc-minimal-data-set-definition). For example, authors should submit the following data:

- The values behind the means, standard deviations and other measures reported;
- The values used to build graphs;
- The points extracted from images for analysis.

Authors do not need to submit their entire data set if only a portion of the data was used in the reported study. If your submission does not contain these data, please either upload them as Supporting Information files or deposit them to a stable, public repository and provide us with the relevant URLs, DOIs, or accession numbers. For a list of recommended repositories, please see https://journals.plos.org/plosone/s/recommended-repositories.

If there are ethical or legal restrictions on sharing a de-identified data set, please explain them in detail (e.g., data contain potentially sensitive information, data are owned by a third-party organization, etc.) and who has imposed them (e.g., an ethics committee). Please also provide contact information for a data access committee, ethics committee, or other institutional body to which data requests may be sent. If data are owned by a third party, please indicate how others may request data access.

Response: Thank you for your constructive feedback regarding the Data Availability Statement. We have revised this section to clearly confirm that our submission contains all raw data necessary to replicate the findings of our study. Specifically, we have included the values behind the means, standard deviations, and other measures reported, as well as the data used to construct graphs. To ensure transparency, we have uploaded the minimal data set as Supporting Information files, which also include relevant metadata and methods. We appreciate your guidance in improving this aspect of our manuscript.

Comment: In the online submission form, you indicated that "The authors confirm that the data supporting the findings of this study are available within the manuscript and its supplementary materials. Furthermore, the corresponding author will be contacted if someone wants to access the data for this study."

All PLOS journals now require all data underlying the findings described in their manuscript to be freely available to other researchers, either 1. In a public repository, 2. Within the manuscript itself, or 3. Uploaded as supplementary information.

This policy applies to all data except where public deposition would breach compliance with the protocol approved by your research ethics board. If your data cannot be made publicly available for ethical or legal reasons (e.g., public availability would compromise patient privacy), please explain your reasons on resubmission and your exemption request will be escalated for approval.

Response: Thank you for your valuable feedback regarding the data availability statement. We appreciate your guidance on this matter. In response to your comment, we have revised the data availability statement as follows: "The authors confirm that the data supporting the findings of this study are available within the manuscript itself and its supplementary materials." We believe this revision clarifies our commitment to ensuring that all data supporting our findings is accessible to other researchers, in accordance with PLOS's policies.

Comment: Please include captions for your Supporting Information files at the end of your manuscript, and update any in-text citations to match accordingly. Please see our Supporting Information guidelines for more information: http://journals.plos.org/plosone/s/supporting-information.

Response: Thank you for your feedback. We have revised the Supporting Information files and included captions for each at the end of the manuscript, as per your guidelines. Additionally, we have updated all in-text citations to ensure they correspond accurately to the new labels. We appreciate your guidance in improving our manuscript.

Point by point response to Reviewer 1's comments

Comment: Thank you for giving me the opportunity to review this manuscript. It was an interesting study to review. My overall view is that the manuscript could be considered for publication subject to addressing my comments below which I hope will improve the quality of the manuscript. There are several grammatical errors which my review doesn't address as I confined my comments to the scientific quality of the manuscript. I will however advise the authors to consider having the manuscript thoroughly proof-read and grammatical errors addressed prior to resubmission.

Response: We would like to acknowledge the reviewer for detailed and constructive comments, which helped us to improve the quality of the manuscript. We have revised the whole manuscript extensively. Below is our point-by-point response to each respective comment

Comment: It would be helpful for the authors to consider justifying their focus on just female fertility in the systematic review (and not male or couple).

Response: While infertility is a complex issue that affects both partners in a couple, this systematic review focuses specifically on the determinants of female infertility in Ethiopia. Research on male infertility in Ethiopia is often limited compared to research on female infertility or couple infertility. Previous studies focused only on female infertility rather than male infertility. This systematic review aims to fill this knowledge gap by synthesizing the available evidence on determinants of female infertility in Ethiopia, contributing to a better understanding of the challenges women face and informing the development of targeted interventions and improved healthcare services. While this review focuses on female infertility, future research should explore the determinants of male infertility and couple infertility in Ethiopia to gain a more comprehensive understanding of the complex factors contributing to infertility in this context."

Additionally, female infertility is often linked to a complex interplay of biological factors, such as hormonal imbalances and reproductive health conditions like polycystic ovary syndrome (PCOS) or endometriosis, making it critical to explore these factors for the development of targeted interventions. The psychological and social impact of infertility on women—including stress, stigma, and discrimination—further underscores the importance of this focus, as it allows for a deeper examination of their unique challenges and better mental health support.

Comment: In the second paragraph please double check reference (5) as the 51.5% refers to primary infertility and not "infertility" as outlined in the manuscript. The sentence after is also

confusing as it states “The burden of female infertility was significantly higher in Sub-Saharan Africa, with primary and secondary infertility accounts 49.91% and 49.79% respectively [3].” However although is uses the word “higher” the figures provided are lower and it draws on a different source (reference 3). This could be made clearer.

Response: thank you very much for positive comment. We have revised this section as per your suggestion.

Comment: More details are required about the previous studies described in paragraph 6 of the introduction described as “fragmented controversial and inconclusive”, justifying this study. The authors should then go on to outline the specific ways their systematic review was going to do to address these issues/gaps and why it was considered important to address these. This is because for a causation systematic review as I have interpreted this study to be, it is advised (https://ebm.bmj.com/content/17/5/137) that at the outset, any hypothesis concerning strength, consistency and temporality should be specified.

Response: Thank you for your insightful comments regarding our manuscript. We appreciate your suggestion to elaborate on the characterization of previous studies as "fragmented, controversial, and inconclusive," as well as the need to outline how our systematic review addresses these issues.

In paragraph 6 of the introduction, we have provided additional details about the inconsistencies found in prior research. “Some studies have reported a strong association between factors like having multiple sexual partner and infertility, while others have not. Similarly, findings on having khat abusive partner, having high alcohol user partner and being rural residency have been contradictory. This lack of consistency makes it challenging to draw clear conclusions about the determinants of female infertility in Ethiopia. This systematic review and meta-analysis aimed to synthesize the available evidence on risk factors for female infertility in Ethiopia to address inconsistencies, evaluate the strength of the associations between specific risk factors and infertility, and examine the consistency of these relationships across various studies. By pooling data from multiple studies, we aimed to provide a more comprehensive and reliable understanding of the key determinants of female infertility in Ethiopia.”

Methods

Comment: Please clarify which authors conducted the literature search and whether it done by two people independently?

Response: Thank you for your valuable feedback. The literature search was conducted independently by two authors, DA and BB, to ensure a comprehensive and unbiased review of the relevant literature. Both authors adhered to a standardized protocol that included predefined keywords and specific inclusion/exclusion criteria. After the initial search, they compared their findings to resolve any discrepancies and ensure consistency in the reviewed literature.

The search was carried out across various databases, including PubMed, African Journals Online (AJOL), ScienceDirect, and the Directory of Open Access Journals (DOAJ). Additionally, studies were sourced from the institutional repositories of Ethiopian universities. The reference lists of all included primary studies were also examined to identify any potentially missed studies. To refine their search techniques and locate pertinent studies, the authors employed the Condition, Context, and Population (CoCoPop) framework, with detailed criteria outlined under the eligibility section.

Comment: What was the process of resolving differences in opinion amongst the authors about studies to include or exclude?

Response: Thank you for your insightful question regarding the resolution of differences in opinion about study inclusion. The process involved several structured steps. Initially, both authors independently reviewed the identified studies based on pre-defined inclusion and exclusion criteria. Following this, they convened to discuss their findings, presenting their rationales for including or excluding specific studies while referencing the established criteria. Through open dialogue, they aimed to reach a consensus, considering factors such as study quality, relevance to the research question, and methodological rigor. In instances where consensus could not be achieved, a third author or external expert was consulted to provide an objective perspective. Ultimately, decisions were made collaboratively, ensuring that both authors agreed on the final list of studies included in the review. This structured approach helped minimize bias and ensured that the review was comprehensive and robust.

Results

Comment: The authors should consider listing the studies in table one in chronological order, with the earliest study listed first. It would also be helpful to clarify the meaning of “facility” and “community based”. Do they mean hospital and community based primary care centres?

Response: Thank you for your insightful feedback regarding Table 1. We appreciate your suggestion to clarify the meaning of "facility" and "community-based" in the table. You are

correct; these terms can be ambiguous. We have addressed your concerns by revising Table 1 in two ways. First, we have listed the studies in chronological order, with the earliest study appearing first. Second, we have removed facility" and "community-based" in the table. We believe these changes improve the readability and understanding of the information presented in Table 1.

Comment: An additional table ("Table 2") summarising the key findings from each of the 6 studies shortlisted would be helpful.

Response: Thank you for your valuable feedback regarding the addition of a table summarizing the key findings from each of the six shortlisted studies. We have created "Table 2," which provides a comprehensive overview of the essential findings, including study design, sample size, significant factors identified, and relevant outcomes. We believe this addition enhances the clarity and accessibility of our results, allowing readers to easily compare the findings across studies. We appreciate your suggestion, which has contributed to improving the quality of our manuscript.

Discussion

Comment: After outlining the findings from their systematic review, the authors should consider discussing their findings in the context of the previous studies described in paragraph 6 of the introduction described as "fragmented controversial and inconclusive". They should clearly outline what value their systematic review has added.

Response: Thank you for your insightful comments regarding the need to discuss our findings in the context of previous studies described as "fragmented, controversial, and inconclusive." We appreciate your suggestion to clearly outline the value added by our systematic review. In our systematic review and meta-analysis, we identified significant associations between female infertility and several risk factors, including having multiple sexual partners, having a khat-abusive partner, and having a high alcohol-user partner. These findings align with some primary studies, reinforcing the idea that these factors are critical determinants of female infertility in Ethiopia.

Conversely, our analysis did not support the significant association of rural residency with infertility, as previously suggested by some studies. The odds ratio (OR = 2.51, 95% CI = -0.37, 5.39) indicates a lack of consistent evidence for this association in our pooled analysis. This

discrepancy highlights the fragmented nature of existing research and emphasizes the importance of our systematic review in synthesizing evidence.

Conclusion

Comment: The authors should consider also including recommendations for future research.

Response: Based on the findings from the systematic review and meta-analysis, several key recommendations for future research emerge. Investigate additional potential risk factors, such as environmental exposures and mental health issues, could provide a more comprehensive view of influences on reproductive health. It is also important to examine the role of male partners in female infertility, particularly regarding their sexual behavior and health status, as this may significantly affect reproductive outcomes. Lastly, qualitative research could offer insights into women's experiences and perceptions related to these risk factors, highlighting barriers to seeking help or adopting preventive measures.
