# Supplementary material for: Factors associated with female infertility in Ethiopia: A systematic review and meta-analysis
Source: PLoS One. 2025 May 16;20(5):e0323181. doi: 10.1371/journal.pone.0323181 (PMC12083798; doi:10.1371/journal.pone.0323181)
Supplement: Attachment — Submitted filename: Point-by-point response.pdf [file pone.0323181.s007.pdf]

Authors' Point-by-Point Response to the reviewers' and editors' Reports

Title: Determinants of female infertility in Ethiopia: a systematic review and meta-analysis

Journal: PLOS ONE

Article type: Systematic review and Meta-analysis

Submission ID: PONE-D-24-14492R2

Point by point response to academic editor's comment

Dear editor,

We would like to express our sincere gratitude for your thoughtful consideration of our manuscript and the valuable suggestions you have provided. Your feedback has been instrumental in enhancing the quality and impact of our work.

Comment 1: We've checked your submission and before we can proceed, we need you to address the following issues: As required by our policy on Data Availability, could you also please ensure your manuscript or supplementary information includes the following information. Please accept our apologies for not sending you this request sooner.

1) A numbered table of all studies identified in the literature search, including those that were excluded from the analyses.
a) For every excluded study, the table should list the reason(s) for exclusion.
b) If any of the included studies are unpublished, include a link (URL) to the primary source or detailed information about how the content can be accessed.

Response: Thank you for your detailed feedback. In response to your request, we have made the following revisions to our manuscript:

We have prepared a numbered table listing all studies identified in the literature search, as requested. This table includes both included and excluded studies, with the reasons for exclusion clearly outlined for each excluded study. For any unpublished studies included in our analysis, we have provided detailed information on how these studies can be accessed, including links (URLs) to the primary sources. This revised table has been included as part of the supplementary material and is referenced in the main manuscript.

We trust that these revisions meet the requirements of the journal's Data Availability policy.

Comment 2: A table of all data extracted from the primary research sources for the systematic review and/or meta-analysis. The table must include the following information for each study:
a) Name of data extractors and date of data extraction
b) Confirmation that the study was eligible to be included in the review.
c) All data extracted from each study for the reported systematic review and/or meta-analysis that would be needed to replicate your analyses.
If data or supporting information were obtained from another source (e.g. correspondence with the author of the original research article), please provide the source of data and dates on which the data/information were obtained by your research group.

Response: We appreciate your thorough review and the opportunity to address the necessary requirements regarding data availability. In response to your request, we have prepared a comprehensive table of all data extracted from the primary research sources included in our systematic review and meta-analysis. This table has been attached as supplementary material and includes the Name of Data Extractors and Date of Data Extraction and eligibility assessment. We believe this supplementary material fulfills your requirements regarding data availability and enhances the transparency of our research. Thank you once again for your constructive feedback.

Comment 3: If applicable for your analysis, a table showing the completed risk of bias and quality/certainty assessments for each study or outcome. Please ensure this is provided for each domain or parameter assessed. For example, if you used the Cochrane risk-of-bias tool for randomized trials, provide answers to each of the signalling questions for each study. If you used GRADE to assess certainty of evidence, provide judgements about each of the quality of evidence factor. This should be provided for each outcome.

Response: thank you for your valuable feedback. Regarding your request for a table showing completed risk of bias and quality/certainty assessments for each study or outcome, we would like to clarify that our systematic review did not involve randomized trials, nor did we employ the Cochrane risk-of-bias tool or the GRADE approach for assessing certainty of evidence in this context.

Instead, our analysis focused on observational studies, which typically do not lend themselves to the same risk-of-bias assessments as randomized trials. We conducted a thorough assessment of study quality using the Newcastle-Ottawa quality assessment instrument, and we have included a summary of this assessment in supplementary material S3 Table.

We believe that our approach adequately addresses the quality of evidence within the scope of our review. However, if you feel that additional clarification or detail is necessary, we would be happy to provide further information or supplementary materials as needed.

Comment 4: An explanation of how missing data were handled.

This information can be included in the main text, supplementary information, or relevant data repository. Please note that providing these underlying data is a requirement for publication in this journal, and if these data are not provided your manuscript might be rejected.

Response: We appreciate the editor's valuable feedback on handling missing data in our manuscript, "Determinants of Female Infertility in Ethiopia: A Systematic Review and Meta-Analysis." We would like to clarify our approach, as missing data was not reported in the primary studies we reviewed.

The studies included in our review did not consistently report missing data or dropout rates, making it difficult to assess the extent of missing information. As a result, we based our analysis solely on the available data in each study. We acknowledge that this limitation may affect the comprehensiveness and reliability of our findings.

To address this, we have added a discussion in the Limitations section at the end of the Discussion, highlighting the potential impact of unreported missing data on our conclusions. We also recommend that future studies on female infertility in Ethiopia include comprehensive reporting on missing data and dropout rates to improve the quality of research and future meta-analyses.

Point by point response to Reviewer 2's comments

Comment: The authors address actual hot topic yet without medical/clinical data significance. Infertility is a complex issue often associated with immune system. The manuscript slides on the surface of infertility disease. The discussion part does not trully stand as a discussion yet rather as a sum up of thorough description. The claim that the female infertility infetility is significantly linked with multiple sexual partners is misleading, it is rather STDs that are mentioned only then after. Alcohol and drug issues are known in general to be a problem concerning sperm quality. The individual parameters linked with sperm quality issues are not discussed.

Response: We sincerely thank Reviewer #2 for their insightful comments and constructive feedback, which have significantly contributed to improving the quality and

depth of the manuscript. We have carefully addressed each point raised, as outlined below:

Lack of Medical/Clinical Data Significance:

We appreciate the reviewer’s observation that infertility is a complex condition, often associated with the immune system. While our manuscript primarily focuses on external factors such as sociodemographic, behavioral, and infectious influences on female fertility, we recognize the importance of incorporating a broader medical and clinical perspective, including immune system-related factors, into the discussion of infertility. However, the primary studies included in this meta-analysis did not assess a comprehensive range of factors, such as immune system function and drug use, which could also contribute to infertility. Consequently, our analysis concentrated on a more limited set of factors, potentially overlooking other significant contributors to female infertility. We have acknowledged this limitation in the revised manuscript under the "Limitations" section.

Discussion Lacking Depth:

We understand the reviewer’s concern that the discussion section reads more as a summary rather than a thorough analysis of the findings. To enhance the depth of the discussion, we have reorganized and expanded this section to better explore the implications of our findings and their relevance to the broader literature on infertility. Specifically, we have discussed potential mechanisms behind the observed associations, such as how each factor influence.

Claim about Multiple Sexual Partners and Infertility:

The reviewer correctly pointed out that the claim linking multiple sexual partners directly to female infertility is misleading. We agree with this observation and have revised the manuscript accordingly. In the revised version, we emphasize that the increased risk of infertility is not due to the number of sexual partners per se but due to the higher likelihood of exposure to sexually transmitted diseases (STDs). We have clarified this distinction in both the Results and Discussion sections to ensure that the association between multiple sexual partners and infertility is properly framed within the context of STD risk.

Alcohol use Affecting Sperm Quality:

The reviewer noted that alcohol use are generally known to negatively impact sperm quality but suggested that specific parameters linked with sperm quality issues were not discussed in the manuscript. We have taken this suggestion into account and expanded the section on alcohol and drug use to include a more detailed discussion of the individual sperm parameters affected by these substances. Specifically, we now address how alcohol and drug use can impact sperm count, motility, morphology, and DNA fragmentation, all of which are crucial to successful fertilization. This additional detail enhances the manuscript's focus on the biological mechanisms involved in infertility.

Finally, we believe these revisions have strengthened the manuscript by offering a more nuanced discussion of infertility, including a deeper exploration of the biological mechanisms involved. We are truly grateful for the reviewer’s valuable feedback, which has been instrumental in guiding these improvements. Once again, thank you for your constructive and thoughtful comments.
