# Supplementary material for: Factors associated with female infertility in Ethiopia: A systematic review and meta-analysis
Source: PLoS One. 2025 May 16;20(5):e0323181. doi: 10.1371/journal.pone.0323181 (PMC12083798; doi:10.1371/journal.pone.0323181)
Supplement: Attachment — Submitted filename: Response to editors and reviewers.pdf [file pone.0323181.s008.pdf]

Authors' Point-by-Point Response to the reviewers' and editors' Reports

Title: Determinants of infertility in couples in Ethiopia: a systematic review and meta-analysis

Journal: PLOS ONE

Article type: Systematic review and Meta-analysis

Submission ID: PONE-D-24-14492R3

Point by point response to Reviewer 3's comments

Comment: Thank you for your dedication and effort in addressing the suggested revisions. Your thoughtful updates have significantly enhanced the clarity, rigor, and overall quality of the manuscript. I appreciate your commitment to improving the work and contributing valuable insights to the field.

Response: We would like to acknowledge the reviewer for detailed and constructive comments, which helped us to improve the quality of the manuscript. Your warm comment and precious time and efforts invested in improving this paper are very much appreciated.

Point by point response to Reviewer 4's comments

We would like to acknowledge the reviewer for detailed and constructive comments, which helped us to improve the quality of the manuscript. Your warm comment and precious time and efforts invested in improving this paper are very much appreciated. Below is our point-by-point response to each respective comment

Comment: I read with great interest the Manuscript titled "Determinants of female infertility in Ethiopia: a systematic review and meta-analysis" (PONE-D-24-14492R2), which falls within the aim of this Journal. In my honest opinion, the topic is interesting enough to attract the readers' attention. Nevertheless, authors should clarify some point and improve the discussion citing relevant and novel key articles about the topic.

Response: We sincerely appreciate your thoughtful feedback and are grateful for your positive assessment of the manuscript's topic. We agree that the discussion section can be further enhanced by incorporating additional relevant and recent literature to provide a more comprehensive analysis. As per your suggestion, we have carefully reviewed and included several key articles published recently on female infertility, particularly those focusing on

Ethiopia and the broader African context. These additional references have helped to clarify and strengthen the discussion, offering a more current perspective on the subject.

Comment: Clarity and Structure of Objectives and Scope: The manuscript has a well-defined objective but could benefit from a more focused research question. Consider specifying how your findings address gaps in the literature beyond descriptive summaries.

Response: Thank you for your valuable feedback. In response to your comment regarding the clarity and focus of the research question, we have revised the objective to specify how our findings go beyond summarizing existing studies. This systematic review and meta-analysis not only synthesize the available evidence on the risk factors for female infertility in Ethiopia but also address the inconsistencies in the literature by quantitatively assessing the strength and consistency of the relationships between key determinants and infertility. By doing so, we aim to provide more robust and conclusive insights that can inform future research and public health interventions. Additionally, we have now explicitly defined the research question

Comment (Methodological Rigor): The systematic review appears thorough, but the inclusion and exclusion criteria could be articulated more explicitly in the main text rather than supplementary materials alone.

Response: Thank you for your helpful comment. The inclusion and exclusion criteria are clearly outlined on pages 5 and 6 of the manuscript. The supplementary materials provide detailed information on the reasons for including or excluding each of the 51 studies identified in the literature search. Including all the excluded studies and their reasons for exclusion in the manuscript would result in nearly 7 additional pages, which could overwhelm readers. We believe that incorporating this level of detail in the main text may detract from the clarity and focus of the manuscript. Therefore, we have chosen to present this information in the supplementary materials to preserve the manuscript's readability and conciseness. We hope this explanation addresses your concern, and we are happy to make any further adjustments if needed.

Comment: Address why certain factors such as immune system function or broader socio-economic determinants were not considered.

Response: Thank you for your thoughtful comment. As our study is a systematic review and meta-analysis based on primary studies, our analysis is inherently limited by the factors that were reported and considered in the included studies. Certain factors, such as immune system function

or broader socio-economic determinants, were not included in our review because they were either not reported in the primary studies or were found to be non-significant in the context of female infertility in primary studies .

We have clarified this in the result section under factors associated with female infertility, explaining that the factors included in our review are those that were consistently studied and reported across the primary studies. We acknowledge that while immune system function and socio-economic determinants are important, their exclusion from our review is due to the lack of consistent reporting or statistical significance in the primary studies. Thank you once again for your valuable feedback, which has helped us clarify these limitations in the manuscript..

Comment (Data Transparency and Availability): While supplementary materials include detailed data, these could be summarized in a more accessible format within the manuscript, especially regarding excluded studies and their reasons for exclusion.

Response: Thank you for your helpful comment. The inclusion and exclusion criteria are clearly outlined on pages 5 and 6 of the manuscript. The supplementary materials provide detailed information on the reasons for including or excluding each of the 51 studies identified in the literature search. Including all the excluded studies and their reasons for exclusion in the manuscript would result in nearly 7 additional pages, which could overwhelm readers. We believe that incorporating this level of detail in the main text may detract from the clarity and focus of the manuscript. Therefore, we have chosen to present this information in the supplementary materials to preserve the manuscript's readability and conciseness. We hope this explanation addresses your concern, and we are happy to make any further adjustments if needed.

Comment: Provide explicit details on how missing data were handled in the included studies, as this may affect replicability.

Response: Thank you for your constructive comment. Missing data were handled with care to ensure the robustness, accuracy, and replicability of our findings. For studies with missing outcome data or incomplete reporting of relevant statistics (e.g., sample size, adjusted odds ratio and 95% confident interval for significant factors), we made efforts to contact the original study authors via email to request the missing information. If we were unable to obtain the missing data after reaching out to the authors, we excluded those specific studies or data points from the meta-analysis, as appropriate. The decision to exclude studies or data points was based on the

extent and nature of the missing data; studies with substantial missing data were excluded entirely, while studies with minor missing data were included, provided the missing data did not critically impact the overall analysis.

Comment (Discussion Depth): The discussion is overly descriptive and does not sufficiently explore the implications of the findings in a broader context or theoretical framework. It should integrate more critical perspectives on how the findings align or contrast with existing research.

Response: Thank you for your valuable feedback. We agree that the discussion could benefit from deeper engagement with the broader context. In response, we have revised the discussion as suggested. We've integrated critical perspectives that link our findings to existing research and theoretical frameworks. By examining how our results align with or differ from previous studies, we've worked to better situate our findings within the larger academic conversation. This revision highlights the significance of our results and addresses potential contradictions or gaps in the current literature. We've also expanded on the theoretical implications and explored how our findings contribute to or challenge established concepts in the field.

Comment: Clarify and differentiate causation from association, especially in sensitive areas such as the linkage between multiple sexual partners and infertility.

Response: Thank you for your valuable feedback. We have revised the section to better clarify the distinction between causation and association, particularly in relation to the linkage between multiple sexual partners and infertility. We have emphasized that while studies indicate a significant association between having multiple sexual partners and infertility, it is not the number of partners per se that directly causes infertility. Rather, the increased risk of exposure to sexually transmitted diseases (STDs), which are more likely with multiple sexual partners, is the underlying factor contributing to reproductive health issues. We have made this distinction clearer to ensure that the relationship is understood as an association, not direct causation.

Comment: To date, lifestyle and psychological stress seem to play a potential detrimental effect of female fertility. Considering that available evidence is not robust enough to draw firm conclusion, I would discuss this topic (authors may refer to: PMID: 29026481; PMID: 28620516).

Response: Thank you for your thoughtful suggestion. We agree with the reviewer's point about the potential detrimental effects of lifestyle and psychological stress on female fertility. However, after reviewing the available primary studies, we found that these variables were not

assessed in the studies included in our analysis. As a result, we did not address this topic in the discussion. That being said, we acknowledge this as a limitation of our review and recognize the need for further research in this area.

Comment (Scientific Soundness and Interpretations): Avoid overgeneralizations, such as directly linking behavioral factors like alcohol and khat use by partners to female infertility without a detailed exploration of biological mechanisms and confounding variables.

Response: Thank you for your insightful comment. We agree with your suggestion and have revised the manuscript accordingly. We have clarified the discussion of behavioral factors like alcohol and khat use by partners, ensuring that we provide a more detailed exploration of the biological mechanisms involved, as well as the potential confounding variables. This revision aims to present a more nuanced interpretation of the relationship between these factors and female infertility.

Comment: Expand on the limitations section to include more critical reflections on heterogeneity and publication bias.

Response: Thank you for your comment. We appreciate the opportunity to address concerns regarding publication bias. To assess the presence of publication bias, we conducted Egger's regression test for the included variables. The results indicated no evidence of publication bias or small study effects for the factors of having a history of multiple sexual partners (p-value = 0.873), STDs (p-value = 0.918), and having an alcohol-abusive partner (p-value = 0.658). These findings suggest that the risk of publication bias in these variables is minimal. However, it is important to note that only two studies were available to assess the pooled effect for maternal age over 35 years and having a khat-abusive partner. Due to this limited number of studies, Egger's regression test was not performed for these two variables. Thus, while publication bias was unlikely for the majority of factors, the small sample size for some variables limits the ability to assess this concern fully for all outcomes.

Comment (Figures and Supplementary Materials): Figures like forest plots are informative but could benefit from annotations summarizing the key takeaways for each factor.

Response: Comment (Figures and Supplementary Materials): Figures like forest plots are informative but could benefit from annotations summarizing the key takeaways for each factor.

Response: Thank you for the suggestion. Rather than in frost plot, Detailed annotations summarizing the key takeaways for each factor are provided in the manuscript, specifically in the "Results" section under the subsection titled Factors Associated with Infertility in Couples in Ethiopia.

Comment: The supplementary materials are extensive but lack integration with the main text, which can make the narrative feel disjointed

Response: Thank you for the feedback. We have made efforts to better integrate the supplementary materials with the main text by including brief descriptions or narrative explanations in the manuscript that reference and connect the supplementary content. We hope this helps create a more cohesive flow throughout the document.

Comment (Ethical Considerations and Impact): While ethical approval is not applicable for a meta-analysis, a brief acknowledgment of ethical considerations in data handling and analysis could enhance transparency.

Response: This meta-analysis has been conducted with careful attention to ethical standards and transparency in data handling, despite the absence of a requirement for formal ethical approval. We ensure that all data included are accurate and reliable by critically evaluating each study's methodological quality and identifying any potential biases. We acknowledge that the original studies included in the meta-analysis must have obtained informed consent from participants, aligning with ethical practices upheld by the original researchers. Additionally, we recognize the potential for publication bias and have transparently reported our criteria for study inclusion, noting any limitations stemming from unpublished studies and their impact on the findings. We also disclose any potential conflicts of interest related to the researchers and funding sources of the original studies to maintain transparency and trust. Finally, the results will be presented honestly, highlighting both strengths and limitations, and discussing their implications for practice and future research.

Comment: Expand the policy implications to address the feasibility of proposed interventions within Ethiopia's healthcare framework.

Response: Thank you for the suggestion. We have revised the policy implications to address the feasibility of the proposed interventions within Ethiopia's healthcare framework, considering factors such as resource availability, infrastructure, and potential challenges to effective implementation.

Comment (Language and Presentation): There are grammatical errors and inconsistent terminology (e.g., "alcohol user" vs. "alcohol abusive partner") that should be standardized.

Response: Thank you for your valuable feedback. We apologize for any inconsistencies in terminology and grammatical errors. We have carefully reviewed the manuscript and

standardized the language throughout. Specifically, we have replaced terms like "alcohol user" and "alcohol abusive partner" with consistent and more precise terminology, such as "individuals with alcohol use disorder" or "alcohol-abusing partners," as appropriate to the context. Additionally, we have corrected any grammatical errors and ensured that terminology is used consistently across the entire manuscript. We believe these revisions enhance the clarity and accuracy of our presentation.

Comment: Simplify overly technical language for broader accessibility without losing scientific precision.

Response: Thank you for your valuable suggestion. We have revised the manuscript to simplify the language while maintaining scientific accuracy. We focused on making the content more accessible to a broader audience by reducing the use of overly technical terms and providing clearer explanations where necessary. We hope these adjustments improve the readability of the manuscript without compromising its scientific integrity. If there are specific sections you'd like us to further revise, please let us know.

Comment (Summary): The manuscript is a valuable contribution to understanding infertility determinants in Ethiopia. However, it requires minor revisions to enhance the depth of analysis, methodological transparency, and integration of findings into broader theoretical and practical contexts. Addressing these issues will strengthen its impact and scholarly rigor.

Response: Thank you for your thoughtful feedback. We appreciate your recognition of the manuscript's contribution to understanding infertility determinants in Ethiopia. In response to your comments, we will enhance the depth of analysis by further elaborating on the methodologies used and providing greater transparency in the approach. Additionally, we will integrate our findings into broader theoretical and practical contexts to strengthen the manuscript's impact and scholarly rigor. We believe these revisions will improve the overall quality of the paper, and we are grateful for your suggestions.

Point by point response to Reviewer 5's comments

Comment: Overall, the study offers an interesting perspective summarizing the available studies investigating human infertility in this East-African Country. However, I think there are several

aspects that require additional improvements and elaboration before this article can be considered for publication.

Response: We would like to sincerely thank the reviewer for the thoughtful and constructive feedback. We greatly appreciate the time and effort you have dedicated to reviewing the manuscript, and your comments have been invaluable in enhancing the quality of this study. Below, we provide a detailed point-by-point response to each of your suggestions and concerns.

Comment: Line numbering may be helpful in the review process.

Response: Thank you for your suggestion. We will add line numbering to the manuscript to facilitate the review process and make it easier to reference specific sections. We appreciate your input and hope this will enhance the clarity and efficiency of the review.

Comment (Title): In my opinion, male factors including addiction to toxic substances abuse can't be formally considered among factors generating (or determinants of) female infertility. Although they are potential contributors to couple infertility, the direct impact on female health and reproductive function has yet to be demonstrated. The title and/or the implant of the review should be elaborated accordingly.

Response: Thank you for your valuable feedback. We understand your concern regarding the inclusion of male factors in the context of female infertility. Based on your suggestion, we have revised the title to better reflect the focus on female-specific determinants while acknowledging the role of partner-related factors, without implying a direct causal relationship between male factors and female infertility.

The revised title is: " **Determinants of Infertility in couples in Ethiopia: A Systematic Review and Meta-Analysis** ". We believe this revision more accurately reflects the focus of the review, and we appreciate your insight in guiding this improvement. Thank you once again for your thoughtful comment.

Comment (Abstract): see title concerning male partner's abuse habits

Response: Thank you for pointing this out. In response to your comment, we have revised the abstract to align with the updated title.

Comment: I'm not sure that infertile patients' (or women's) age can be called "maternal" age. If those patients are unable to conceive, they should not be called "mothers", it may sound not appropriate. Distinction between women who had already delivered and who did not (primary and secondary infertility) is not considered.

Response. Thank you for your insightful suggestion. We understand your concern regarding the use of the term "maternal age" for women experiencing infertility. However, the primary studies included in our systematic review and meta-analysis consistently reported the term "maternal age" in relation to their study participants, regardless of whether they had previously delivered. These studies primarily assessed risk factors for general infertility without making a separate distinction between primary and secondary infertility.

Given that our study is a systematic review and meta-analysis of primary studies, we have opted to use the same variable name, "maternal age", as reported in the original studies to maintain consistency with their terminology.

Regarding the distinction between primary and secondary infertility, we appreciate your point. The included primary studies did not differentiate the risk factors for primary and secondary infertility separately, and thus, our review reflects the overall infertility risk factors rather than making this distinction. We hope this explanation clarifies the rationale for using the term "maternal age" and addressing infertility risk factors as reported in the primary studies. Thank you again for your valuable feedback.

Comment (Introduction): We agree that infertility has a substantial social and economical impact on women's life. However, when the authors emphasize that this burden is higher in SubSaharan Africa and even higer in Ethiopia, these claims are not supported by quantitative investigations. Prevalence studies cited above have shown similar rates. By contrast, this paragraph emphasizes specific features and weaknesses of this context, that still provide useful information to understand the situation in low- and middle-income countries. Please, rephrase.

Response: Thank you for your thoughtful comment. We have revised the paragraph to focus more on the specific challenges and contextual factors that contribute to the burden of infertility in low- and middle-income countries, particularly in Ethiopia. We have removed the comparison implying that the burden is definitively higher in Sub-Saharan Africa and Ethiopia, and instead highlighted the unique social, economic, and healthcare-related factors that exacerbate the situation in these regions. We hope this revision better reflects the complexities of infertility in these contexts. Thank you again for your valuable feedback.

Comment (Methods): Women reporting infertility, which could be "couple infertility" given that male factors are significantly correlated, is not exactly synonymous with female infertility.

This assumption potentially undermines the clarity of the results presented and requires more clarification in methods and discussion.

Response: Thank you for your valuable feedback. We understand your concern regarding the inclusion of male factors in the context of female infertility. You are correct that infertility in couples can be influenced by both female and male factors, and it is important to clarify that our study specifically focuses on female infertility.

In response to your suggestion, we have revised both the title and the methodology section to better reflect the scope of the review, which focuses on female-specific determinants while acknowledging the role of partner-related factors, without implying a direct causal relationship between male factors and female infertility.

The revised title is: "Determinants of Infertility in couples in Ethiopia: A Systematic Review and Meta-Analysis ". Additionally, we have clarified in the Methods section that the studies included in our review primarily assessed female infertility, but some studies also considered partner factors. We have made it clear that while male factors may influence infertility in couples, this review specifically examines female infertility and the associated female and partner-related risk factors. We hope these revisions help address your concern and improve the clarity of the manuscript. We appreciate your insightful feedback, which has guided us in making these important clarifications. Thank you once again for your constructive comment. We believe this revision more accurately reflects the focus of the review, and we appreciate your insight in guiding this improvement. Thank you once again for your thoughtful comment.

Comment: There's no distinction between primary and secondary infertility. Therefore, I believe that more caution is required when drawing conclusions and recommendations in the main text, abstract and conclusions.

Response: Thank you for raising this important point. We appreciate your feedback regarding the distinction between primary and secondary infertility. You are correct that the primary studies included in our review did not make a clear distinction between the risk factors for primary and secondary infertility, and thus, our analysis reflects the overall infertility risk factors without differentiating between the two.

To address your concern, we have taken extra caution in the Discussion, Abstract, and Conclusions sections. We now emphasize that the findings from this review are based on studies that combined both primary and secondary infertility cases and therefore may not reflect the

specific risk factors for either group. We have added a note that the generalizability of the findings to primary versus secondary infertility should be interpreted with caution, given the lack of differentiation in the included studies. We hope this clarifies the potential limitations in the conclusions and ensures that the recommendations are presented with the appropriate level of caution. Thank you once again for your insightful feedback, which has helped improve the accuracy and clarity of the manuscript.

Comment (Results): Fair presentation of results is provided.

Response: Thank you for your positive feedback. We are glad that the presentation of the results meets your expectations. We appreciate your careful review and are pleased that the data is presented clearly.

Comment (Discussion): The claim that chlamydia infection increases the risk of endometriosis is not supported by the available literature. In fact, adequate references are not provided; indeed, there is evidence suggesting the absence of correlation*. However, both endometriosis and pelvic infections may generate pelvic adhesions, contributing to infertility. By contrast, evidence aggregating several studies with different design should be discussed with caution**

Response: Thank you for the comment. We have revised the statement to better align with the available literature. Specifically, we have removed the claim that chlamydia infection increases the risk of endometriosis and acknowledged the evidence suggesting the absence of a direct correlation.

Comment: Minor issues related to the English language require to be addressed.

Response: Thank you for pointing this out. We have made the necessary revisions to address the minor language issues and improve the overall clarity and readability of the manuscript.

Comment: Please declare eventual use of artificial intelligence support.

Response: Thank you for the comment. We have addressed the use of artificial intelligence support by acknowledging the AI tools in the "Declaration" section, under the sub-section titled Acknowledgment.
