# Supplementary material for: Factors associated with female infertility in Ethiopia: A systematic review and meta-analysis
Source: PLoS One. 2025 May 16;20(5):e0323181. doi: 10.1371/journal.pone.0323181 (PMC12083798; doi:10.1371/journal.pone.0323181)
Supplement: Attachment — Submitted filename: Response_to_editors_and_reviewers_auresp_4.pdf [file pone.0323181.s009.pdf]

<u>Authors' Point-by-Point Response to the reviewers' and editors' Reports</u>
Title: Factors associated with female infertility in Ethiopia: a systematic review and meta-analysis
Journal: PLOS ONE
Article type: Systematic review and Meta-analysis
Submission ID: PONE-D-24-14492R3

<u>Point by point response to Reviewer 4's comments</u>

Comment: I carefully evaluated the revised version of this manuscript. Authors have performed the required changes, improving significantly the quality of the paper.
Response: Dear Dr. Pietro Serra, we sincerely appreciate your detailed and constructive feedback, which has greatly enhanced the quality of our manuscript. Your kind words, time, and effort in improving this paper mean a lot to us. Thank you again for your positive feedbacks.

<u>Point by point response to Reviewer 5's comments</u>

Dear Dr. Michele Orsi,
We would like to acknowledge you for detailed and constructive comments, which helped us to improve the quality of the manuscript. We really appreciate your insightful critique, which has significantly strengthened the accuracy, clarity, and methodological rigor of our work. Thank you again for guiding us toward a more precise and scientifically sound presentation of our findings. Blow is our point-by-point response to each respective comment.

Comment: I think the authors have made a commendable effort to improve their manuscript. However, some aspects require further revision to meet the standards of this Journal. Thanks for adding line numbering.

Response: We are grateful for the reviewer's thoughtful feedback and acknowledgment of our efforts to improve the manuscript. We have carefully addressed the remaining concerns to ensure the manuscript meets the journal's standards. Please find our detailed responses to each comment below.

Comment: Line 1, 27, 37, 42, 84, 86, 120, 122, 226, 283, 290 etc. - I apologize if my previous comment was not entirely clear when I stated: "In my opinion, male factors including addiction to toxic substances abuse can't be formally considered among factors generating (or determinants of) female infertility. Although they are potential contributors to couple infertility, the direct impact on female health and reproductive function has yet to be demonstrated. The title and/or the implant of the review should be elaborated accordingly." The criticism was not intended for the purpose of changing "female infertility" to "couple infertility," while leaving the research criteria unchanged and focused on the female condition. In my opinion, this change is not

acceptable, as it is not possible to title and discuss a paper focused on “couple infertility,” while the search criteria remained the previous ones, that is, referring to factors related to “female infertility.”

Rather, my critique was aimed at discussing the other part of the sentence, i.e., “determinants” of female infertility, either in the title or in the abstract and text. this statement suggests that male factors (particularly partners who abuse alcohol or chat) are determinants, or con-causal, or risk factors, for female infertility. This assumption is formally incorrect. They are associated factors, which means they are detected in women evaluated for infertility by questionnaires, and could certainly contribute to couple infertility, but not directly generate/cause female infertility. In fact, cross-sectional or retrospective studies cannot determine causal associations. In summary, it remains a paper of potential interest. However, all the changes made to rename “female” infertility to “couple” infertility should be reinstated (according to the research criteria). Title and findings must meet the methodological standard of the studies included as well as clinical rationale, i.e., “factors associated with female infertility, which differs from "risk factors" or "determinants" or "causal factors".

Response: Thank you for your valuable and detailed comment. We sincerely appreciate your clarification regarding the distinction between "determinants" and "associated factors" in the context of female infertility. Initially, we did not place sufficient emphasis on the term "determinants" and used it in the title and text without fully considering its implications. We now understand that the term "determinants" implies a causal relationship, which is not supported by the cross-sectional nature of the primary studies included in our analysis.

We fully agree with your observation that male factors, such as addiction to toxic substances (e.g., alcohol or khat), cannot be formally considered determinants or causal factors of female infertility. While these factors may contribute to couple infertility, their direct impact on female reproductive health and function has not been demonstrated. Instead, these factors should be categorized as associated factors, as they were identified through questionnaires in cross sectional studies and may correlate with infertility but do not establish causation.

In line with your feedback, we have revised the manuscript to replace "determinants" **with "factors associated with female infertility in Ethiopia**." This adjustment ensures that the terminology aligns with the methodological standards of the included studies and the clinical rationale. Importantly, this change did not affect the search criteria, research framework, eligibility criteria, the total number of included and excluded studies, methodology and discussion of the study. We have also reinstated the focus on "female infertility" throughout the manuscript, as the search criteria and research framework were specifically designed to address factors associated with female infertility.

Comment: Line 308 - The main risk factor for STD is having multiple partners! This aspect can't be missed in the discussion. The two conditions are most likely related.

Response: We sincerely appreciate your valuable feedback regarding the relationship between having multiple sexual partners and the risk of sexually transmitted disease (STDs). We would like to clarify that the association between having multiple sexual partners and female infertility was already discussed in Lines 304–314, and the association between multiple sexual partners and STDs was addressed in Lines 308–313. Additionally, the association between STDs and female infertility was thoroughly discussed in Lines 306–332. These sections collectively highlight the interconnected nature of these risk factors and their impact on female infertility. We believe these discussions adequately address the relationship between multiple sexual partners, STDs, and infertility as raised in your comment.

Comment: "Maternal age" – Even though I respect the authors attitude to be adherent to previous publications, I would have made the same comments to those papers. Additionally, one of the publications included in the review specially refers to primary infertility. How can we name as "mother" someone who has never had the chance to conceive? Couldn't someone potentially feel offended?

Response: Thank you for your insightful comment. We understand and appreciate your concern regarding the term "maternal age," particularly in the context of individuals who have not had the opportunity to conceive. Our intention in using this term was to maintain consistency with the primary studies we referenced. However, we acknowledge that this terminology may not be fully inclusive, especially for individuals experiencing primary infertility.

To address this, we carefully reviewed our manuscript and replaced "**maternal age**" with more inclusive alternatives, such as "women's age," through out the document. We believe these terms more accurately reflect the broader population under discussion without assuming prior conception or motherhood. This adjustment not only enhances the inclusivity of our work but also ensures greater precision in describing our study cohort.

Comment: Heterogeneity - Isn't the moderate to high heterogeneity of the included studies worth discussing?

Response: We sincerely appreciate your valuable feedback regarding the heterogeneity observed in the included studies. In response to your comment, we have explicitly mentioned the presence of moderate to high heterogeneity under each associated factor in the forest plots and discussed its implications in the Discussion section. Additionally, we have acknowledged heterogeneity as a limitation in the Limitations section, highlighting its potential impact on the generalizability of the findings. These revisions provide a comprehensive discussion of heterogeneity and its relevance to the interpretation of our results.

Comment: Line 228-229, 374-375 – Please add hormonal factors to this list, at least in general, (i.e. we all know thyroid disorders, premature ovarian failure, etc.) as well as appropriate citations from international population-based reviews for this list. And what about cigarette smoking?

Response: We sincerely appreciate your valuable feedback. In response to your comment, we have revised the manuscript to explicitly include hormonal factors (e.g., thyroid disorders, premature ovarian failure, hyperprolactinemia, and polycystic ovary syndrome) and cigarette smoking in the list of associated factors that were not comprehensively assessed in the primary studies. These factors are now mentioned in Lines 228–229 and 374–375.

Comment: Line 352-354 - Please add appropriate references

Response: Thank you for your valuable feedback. In response to your comment, we have revised Lines 352–354 and added appropriate references to support the statement. The revised text now includes citations from relevant studies that address the decline in egg quality and its impact on fertility with advancing maternal age.

Comment: Line 377-379 – The need of additional investigations about those factors just mentioned above in the Ethiopian population, as well as the need to invest significant resources to understand the impact of these conditions on infertility as a public health issue are among the main implications of the findings. Therefore, in my opinion they should be included in that paragraph more than just after limitations, as well as in conclusions and abstract!

Response: We sincerely appreciate your valuable feedback. In response to your comment, we have incorporated the understudied factors (e.g., hormonal imbalances, lifestyle, and socio-economic determinants) and recommendations for future research into the implications section, abstract, and conclusion section of the manuscript, as per your suggestion. These revisions emphasize the need for additional investigations into these factors and highlight the importance of investing resources to address infertility as a public health issue in Ethiopia. We believe these changes strengthen the manuscript and align it more closely with the public health priorities of the Ethiopian population.

Comment: Limitations – Was the separation between primary and secondary infertility not feasible for this study? This is a potential limitation as well as a relevant additional clinical implication of the findings. Additionally, an emphasis on this topic in the conclusions would also be worthwhile, helping as mentioned above to dictate future research directions essential to improve the understanding of this important public health issue.

Response: We sincerely appreciate your valuable feedback regarding the separation between primary and secondary infertility. In response to your comment, we acknowledge that the separation between primary and secondary infertility was not feasible for this study due to the

lack of data in the primary studies. However, we recognize this as an important limitation and have emphasized its relevance in the Limitations section. Additionally, we have highlighted the need for future research to distinguish between primary and secondary infertility to improve the understanding of this important public health issue and inform targeted interventions under conclusion section.
