# Supplementary material for: Factors associated with female infertility in Ethiopia: A systematic review and meta-analysis
Source: PLoS One. 2025 May 16;20(5):e0323181. doi: 10.1371/journal.pone.0323181 (PMC12083798; doi:10.1371/journal.pone.0323181)
Supplement: Attachment — Submitted filename: Response_to_editors_and_reviewers_auresp_5.pdf [file pone.0323181.s010.pdf]

Authors' Point-by-Point Response to the reviewers' and editors' Reports

Title: Factors associated with female infertility in Ethiopia: a systematic review and meta-analysis

Journal: PLOS ONE

Article type: Systematic review and Meta-analysis

Submission ID: PONE-D-24-14492R4

Point by point response to editor comments

Comment: Please review your reference list to ensure that it is complete and correct. If you have cited papers that have been retracted, please include the rationale for doing so in the manuscript text, or remove these references and replace them with relevant current references. Any changes to the reference list should be mentioned in the rebuttal letter that accompanies your revised manuscript. If you need to cite a retracted article, indicate the article's retracted status in the References list and also include a citation and full reference for the retraction notice.

Response: Dear Assoc. Prof. Phakkharawat Sittiprapaporn and the PLOS ONE Editorial Team,

Thank you for your note regarding the reference list. We have carefully reviewed all cited references in our manuscript and confirm that they are complete, correct, and free of retracted papers. Should any further adjustments be required, we are happy to address them promptly.

Point by point response to Reviewer 5's comments

Dear Dr. Michele Orsi,

We would like to acknowledge you for detailed and constructive comments, which helped us to improve the quality of the manuscript. We really appreciate your insightful critique, which has significantly strengthened the accuracy, clarity, and methodological rigor of our work. Thank you again for guiding us toward a more precise and scientifically sound presentation of our findings. Blow is our point-by-point response to each respective comment.

Comment: The last two comments:

- I appreciate they embedded the need of exploring the impact of additional factors in the discussion, conclusion and abstract. However, to make the abstract and conclusions clearer and straightforward, removal of details may be considered "(e.g., thyroid disorders, premature ovarian failure, hyperprolactinemia, and polycystic ovary syndrome)". The list is already detailed in my opinion. "Gynecological conditions" may be the only element to add to the list, but details as for endocrinological conditions may be added to the discussion paragraph (e.g. uterine fibroids, endometrial polyps, endometriosis and adenomyosis).

- In the discussion paragraph, where the authors highlight those additional factors that need to be explored in future literature, detailed references to justify that claim should be added. We are aware that the review is not focused on those factors in different populations. However, the discussion should be justified by the existing literature, and potentially enriched by findings in optimally-resourced settings. Examples are provided below, and should be followed for all the conditions mentioned according to previous studies, in both similar and different settings.

Response: We sincerely appreciate the reviewer's thoughtful feedback, which has helped strengthen our manuscript. In response to the final comments, we have streamlined the abstract and conclusions by removing the detailed listing of specific conditions (e.g., thyroid disorders, premature ovarian failure) and replacing them with the broader terms "gynecological and endocrinological conditions" to improve clarity and readability. The specific examples mentioned by the reviewer, including uterine fibroids, endometrial polyps, endometriosis and adenomyosis, have been incorporated into the discussion section where they can be properly contextualized. Regarding the discussion of factors needing further exploration, we have carefully reviewed the literature and added relevant references from both optimally-resourced settings and comparable populations to better substantiate our claims. These citations provide appropriate justification for highlighting these areas as needing further research while maintaining our focus on the study's primary objectives. We believe these revisions have significantly improved the manuscript.
